# Associations between sensitization to perennial/seasonal allergens and childhood asthma 

**DOI:** 10.5414/ALX01882E

**Published:** 2018-09-01

**Authors:** M. Kuzdak, J. Jerzynska, I. Stelmach, D. Podlecka, P. Majak, A. Janas, R. Stelmach, W. Stelmach

**Affiliations:** 1 Department of Social and Preventive Medicine, Social Medicine Institute, Medical University of Lodz,; 2 Department of Pediatrics and Allergy, Medical University of Lodz, Copernicus Memorial Hospital,; 3 Institute of Dental Surgery, Faculty of Medicine and Dentistry, Medical University of Lodz, Poland

**Keywords:** sensitization, seasonal allergens, perennial allergens, allergic rhinitis, asthma

## Abstract

Background: Childhood asthma is an important public health problem worldwide. Risk factors for asthma development include allergic sensitization and exposure to animals. Objective: To identify which (perennial or seasonal) inhalant allergens are associated with asthma and allergic rhinitis in children. Methods: This was a cross-sectional, retrospective study. We evaluated data from medical documentation of 6,000 children (aged 6 – 18 years) with diagnosed asthma and/or allergic rhinitis who had attended our allergy outpatient clinic. Into the analyses we included those subjects who had specific IgE test done during diagnostic procedures to confirm allergen sensitization. Results: We included 5,076 children in the analysis. We showed that among seasonal allergens only sensitization to timothy or birch significantly changed the prevalence of allergic rhinitis and asthma diagnosis. Of the perennial allergens, house dust mite or cat were most closely related with both allergic rhinitis and asthma. Results of ROC curve analysis showed that in atopic children the specific IgE level of seasonal allergens did not significantly change the prevalence of asthma diagnosis. Sensitization to more than one perennial allergen significantly increased the prevalence of allergic rhinitis and asthma. Conclusion: We showed that sensitization to the seasonal allergens timothy and birch as well as to the perennial allergens house dust mite and cat, is associated with asthma and allergic rhinitis in children. Our study determined the role of multiple perennial indoor allergens in the developement of allergic diseases in children. The identification of the specific allergens makes them potential targets for intervention and prevention strategies.

**German version published in Allergologie, Vol. 40, No. 1/2017, pp. 23-28**

## Introduction 

Childhood asthma is an important public health problem worldwide [1]. Risk factors include genetic, environmental, and host factors. Common risk factors are: sex, socioeconomic status, exposure to tobacco smoke [2], exposure to air pollution [3], diet [2, 4], obesity [5], allergic sensitization, family structure, and exposure to animals [2] as well as a family history of atopic diseases [2, 6]. To date, most research has focused on the association of asthma with multiple sensitizations, but the type and number of allergen sensitization are unknown [7]. Few studies have investigated home environmental exposures at school-age as putative risk factors for asthma development in adolescence [8]. However, this may be highly relevant in childhood, where a wide range of total serum or specific IgE (sIgE) levels is often detected. 

We aimed to find out the sensitization to which inhalant allergens (perennial or seasonal) was associated with asthma and allergic rhinitis in children. 

## Materials and Methods 

**Study design**

This was a cross-sectional, retrospective study. We evaluated data from medical documentation of 6,000 children (aged 6 – 18 years) with physician-diagnosed asthma and/or allergic rhinitis who had attended our allergy outpatient clinic between January 2005 and December 2012. Into the analyses we included those subjects who, during diagnostic procedures, had specific IgE test done to confirm allergen sensitization. Confirmation of asthma/allergic rhinitis diagnosis was made previously by the allergists according to the standard definitions of diseases in the latest guidelines [9, 10] or their respective previous versions (due to the fact that patients had been admitted since January 2005). The retrospective analysis of the study for the evaluation of data from medical documentation of children was approved by the Medical Ethics Committee of the Medical University of Lodz. The study was registered on: www.ClinicalTrials.gov, with ClinicalTrials.gov ID: NCT01805635. 

**Allergen sensitization**

Allergen sensitization was defined as specific IgE of ≥ 0,35 KU/L for at least one of the tested allergens (chemiluminescence method (CLIA), Immulite 2000, XPI, Siemens, Germany). For purpose of the study, we defined perennial allergy as presence of serum IgE of ≥ 0,35 KU/L specific for the perennial allergens dust mites, molds, cat and dog dander; seasonal allergy was defined as presence of serum IgE of ≥ 0,35 KU/L specific for the seasonal allergens grasses, wild grasses, and tree pollen. 

## Statistical methods 

The associations between the diagnosis allergic rhinitis or asthma and the serum level of specific IgE were analyzed using univariate followed by multivariate logistic regression. Cut-off points with optimal discrimination (the highest sensitivity and specificity) of specific IgE levels were calculated in ROC curve analysis. All statistical analyses were performed using StatSoft Statistica for Windows, release 8.0 (StatSoft, Inc., Tulsa, USA). p < 0.05 was used as a definition of statistical significance. 

## Results 

We included 5,076 children in the analysis (absolute number of patients who had specific IgE results). Clinical characteristics are given in Table 1. 

In the first step we implemented logistic regression analysis to find out the sensitizing allergens most closely related to allergic rhinitis/asthma. Analyses for perennial and seasonal allergens were made independently. The results of the multivariate models are presented in Table 2. We showed that among the seasonal allergens, only sensitization to timothy or birch significantly changed the prevalence of allergic rhinitis and asthma diagnosis (Table 2). Among the perennial allergens, house dust mite or cat were most closely related to both allergic rhinitis and asthma in our population (Table 2). 

In the next step we assessed the relationship between specific IgE level and the diagnosis of allergic rhinitis/asthma among atopic children. Area under the receiving operating characteristic (ROC) curve for serum levels of sensitizing allergens as discrimination threshold for allergic rhinitis and asthma diagnosis are given in Table 3 and Table 4, respectively. Results of the ROC curve analysis showed that among atopic children, specific IgE levels of seasonal and perennial allergens do not significantly change the prevalence of asthma diagnosis. The cut-off levels of specific seasonal and perennial IgE with optimal diagnostic accuracy are given in Table 3 and Table 4. 

Finally we checked for the cumulative effect of multiple allergens. As was expected, sensitization to more than one allergen significantly increased the prevalence of allergic rhinitis and asthma diagnosis. However, this phenomenon was visible only for perennial allergens (Table 5). 

## Discussion 

To date, only few studies have considered polysensitization as a risk factor for asthma development in prediction models and those were performed in populations from affluent countries. We aimed to find out the sensitization to which inhalant allergens (perennial or seasonal) was associated with asthma and allergic rhinitis in a population of Polish children. Our findings showed that only *Dermatophagoides farinae,* cat dander, birch, and *Timothy grass* allergens were associated with a diagnosis of asthma and allergic rhinitis. We determined the cut-off levels of specific seasonal and perennial IgE with optimal diagnostic accuracy. 

Our results cannot be compared to other studies due to the lack of studies with similar methodology. Kihlström et al. [11] found that exposure to birch pollen in early infancy may increase the risk of atopic diseases in childhood. Other authors state that persistent pollen exposure in early life appears to increase the risk of asthma and hay fever in children [7, 12, 13, 14]. This difference to our study may be due to the fact that these assessments were made in completely different populations and that we have not examined the effect of family history by season of birth. 

Our results suggest that among perennial allergens, house dust mite and cat were most closely related with both allergic rhinitis and asthma in our population. Our results are similar to Maheswaran et al.’s [15], who confirmed that early exposure to dust mite allergens is a risk factor for atopic asthma and bronchial hyperresponsiveness in adolescents. In two large population-based cohort studies from Germany and South England [16, 17], no significant association was found between early pet exposure and asthma at the age of 5 – 7 years. There are several studies discussing the idea that early exposure to furry pets may protect children from later allergy [18, 19, 20]. 

We found that among atopic children, the level of specific IgE to seasonal allergens does not significantly change the prevalence of the diagnosis of childhood asthma. Contradictory, Chiu et al. [21] report a significantly increased risk for the development of asthma and rhinitis in children with sensitization to inhalant allergens after the age of 2 years. This discrepancy may be due to a relatively small sample size in Chiu et al.’s research as compared to our study. 

Investigating the cumulative effect of multiple allergens, we found that sensitization to more than one perennial allergen significantly increases the prevalence of allergic rhinitis and asthma. On the contrary, Agache and Ciobanu [22] showed that polysensitization to seasonal pollens was a risk factor for asthma in children with seasonal allergic rhinitis. 

The main limitation of our study was its retrospective design. We evaluated data from the patients’ medical documentation, which could have partly influenced the accuracy of our results. Another limitation is a relatively wide range of the study subjects’ age (from 6 – 18 years). However, age did not change the goodness-of-fit of the multivariate models. Therefore, subgroup analyses were less reasonable. We evaluated data from the medical documentation of patients from an allergy clinic; thus, it is possible that our study population was skewed. However, all patients participating in this study were under regular care of allergy specialists from our clinic, including physical examination, lung function measurements, and other necessary tests, which exclude any doubts concerning the heterogeneity of diagnostic procedures. 

In conclusion, we showed that sensitization to the seasonal allergens timothy or birch as well as to the perennial allergens house dust mite or cat is associated with asthma and allergic rhinitis in children. Our study determined the role of multiple perennial indoor allergens in the development of allergic diseases in children. The identification of the specific allergens makes them potential targets for intervention and prevention strategies. Thus, our findings could be an additional reason to consider an early introduction of immunotherapy in children sensitized to the above allergens. 

**Table 1.** Characteristics of the study population.

**Variable**	**N**	**%**
Age [years], mean(SD)	10.4 (4.1)	
Male gender	2,877	56.7
Diagnosis of allergic rhinitis	1,192	23.5
Diagnosis of asthma	1,480	29.2
Presence* of specific IgE in serum:		
HDM1	999	19.7
HDM2	987	19.4
Alternaria	451	8.9
Cladosporium	20	0.4
Cat	498	9.8
Dog	83	1.6
Timothy	1,057	27.7
Dactylis glomerata	679	17.8
Rye	946	24.8
Birch	774	20.3
Alder	219	5.7
Hazel	184	4.8
Mugwort	339	8.9

*Specific IgE > 0,15 IU/L.

**Table 2.** Associations between allergy profile and diagnosis of allergic rhinitis/asthma. Data is presented with OR (95%CI) as a result of multivariable logistic regression analysis; only statistically significant predictors of allergic rhinitis/asthma are given (final models).

	**OR** *^a^*	**95% CI**		**p**	**OR** *^b^*	**95% CI**		**p**
**Seasonal allergens**								
Timothy	1.012	1.008	1.016	<0.0001	1.011	1.007	1.014	< 0.0001
Birch	1.009	1.004	1.013	0.0003	1.006	1.001	1.010	0.0176
**Perennial allergens**								
House dust mite	1.011	1.006	1.015	<0.0001	1.014	1.010	1.018	< 0.0001
Cat	1.014	1.008	1.021	<0.0001	1.009	1.003	1.016	0.0037

^a^Dependent variable – diagnosis of allergic rwhinitis; ^b^dependent variable – diagnosis of asthma.

**Table 3.** Area under the ROC curve for serum levels of sensitizing allergens as discrimination threshold for the diagnosis of allergic rhinitis.

**Serum level of sensitizing allergens**	**Area** ^a^	**95% CI**		**p-level** ^b^	**Cut-off** ^c^
House dust mite	0.59	0.56	0.62	< 0.0001	5.20
Cat	0.57	0.52	0.61	0.0031	12.60
Birch	0.58	0.54	0.62	0.0001	8.90
Timothy	0.56	0.53	0.60	0.0001	6.00

^a^Area under the ROC curve; ^b^null hypothesis: true area = 0.5; ^c^sIgE level [IU/L] with highest area under the ROC curve.

**Table 4.** Area under the ROC curve for serum levels of sensitizing allergens as discrimination threshold for the diagnosis of asthma.

**Serum level of sensitizing allergens**	**Area** *^a^*	**95% CI**		**p-level** *^b^*	**Cut-off** *^c^*
House dust mite	0.61	0.58	0.65	< 0.0001	9.90
Cat	0.55	0.50	0.59	0.0332	12.60
Birch	0.53	0.49	0.57	0.1347	4.50
Timothy	0.52	0.48	0.55	0.3350	2.50

^a^Area under the ROC curve; ^b^null hypothesis: true area = 0.5; ^c^sIgE level [IU/L] with highest area under the ROC curve.

**Table 5.** Association between the number of perennial and seasonal sensitizing allergens and the diagnosis of allergic rhinitis.

**Number of sensitizing allergens:**	**No AR**		**AR**		**OR** *^a^*	**95% CI**		**p**
	**N**	**%**	**N**	**%**				
**Perennial**								
Non	3,003	77.3	575	48.2				
One	306	7.9	173	14.5	ref			0.0069
Two	362	9.3	258	21.6	1.26	0.99	1.61	0.0644
Three and more	213	5.5	186	15.6	1.54	1.18	2.03	0.0017
**Seasonal**								
Non	1,918	71.8	372	40.6				
One	191	7.1	109	11.9	ref			0.0644
Two	167	6.3	121	13.2	1.27	0.91	1.77	0.1585
Three and more	396	14.8	315	34.4	1.29	0.96	1.84	0.0592

^a^Dependent variable – diagnosis of allergic rhinitis; ref – reference category; AR = allergic rhinitis.
